# C-756-04. Institutional Framework to Improve Physical and Occupational Therapy Engagement in Pediatric Patients with Burn Injury

**DOI:** 10.1093/jbcr/irag033.048

**Published:** 2026-04-06

**Authors:** Nomongo Dorjsuren, Emmanuel Giannas, Anvith Reddy, Kara Arps, Annalesa Sackey, Holly Heavner, Jessica H Ballou, Robel Beyene, Anne L Lambert Wagner, Elizabeth L Dale Slater

**Affiliations:** Vanderbilt University Medical Center, Nashville, TN; Vanderbilt Burn Center, Nashville, TN; Vanderbilt University Medical Center, Nashville, TN; Vanderbilt University Medical Center, Nashville, TN; Vanderbilt University Medical Center, Nashville, TN; Vanderbilt University Medical Center, Nashville, TN; Vanderbilt University Medical Center, Nashville, TN; Vanderbilt University Medical Center, Nashville, TN; Vanderbilt University Medical Center, Nashville, TN; Vanderbilt University Medical Center, Nashville, TN

## Abstract

**Introduction:**

A multidisciplinary team (MDT) approach, including early physical and occupational therapy (PT/OT), is crucial in managing acute pediatric burn injury. However, PT/OT involvement can be delayed by gaps in institutional processes and communication between burn and therapy providers. This quality improvement study implemented an institutional framework aiming to increase timely PT/OT engagement following acute burn admission in pediatric patients.

**Methods:**

A retrospective cohort study was performed in a single tertiary pediatric academic center. A multidisciplinary institutional framework was initiated on July 1st, 2024, and applied to all acute pediatric burn patients. This included acquisition of a burn-specialized advanced practice provider, relocation of pediatric burn patients from the adult burn unit to the children’s hospital, protocolization of PT/OT orders at admission, and the introduction of an educational handbook for burn residents. The post-intervention cohort was compared to a historical cohort of patients admitted eight months prior to framework implementation. Federal holiday admissions without PT/OT staffing were excluded. The primary outcome measure was the percentage of patients who did not have PT/OT orders placed during their hospital stay. The secondary outcome measure included time to first PT/OT evaluation.

**Results:**

A total of 138 patients were included. The cohort was 70% male and 87% non-Hispanic, with a median interquartile range (IQR) age of 5 (1.5-13) years old and median (IQR) TBSA of 7 (4-13) %. Most burns were partial thickness (64%), primarily caused by scald (43%) or flame (43%) injuries. Baseline demographics and injury characteristics including age, sex, TBSA, burn thickness, and injury mechanism, were similar between pre- and post-intervention cohorts. Post-intervention, the proportion of patients without a PT/OT order declined from 21% to 1.6% (*p*=.001); after excluding observation-only admissions, rates declined from 14.4% to 0% (*p*=.002). The proportion of PT/OT evaluations completed within 24 hours of admission trended upwards from 79% to 84% (*p*=.6) after intervention.

**Conclusions:**

MDT structural changes improved consistency of PT/OT evaluations among pediatric burn patients. Implementing institutional frameworks that protocolize communication between burn and therapy teams may improve early rehabilitation access, a vital aspect of acute burn management in pediatric patients.

**Applicability of Research to Practice:**

Embedding burn-specific expertise, optimizing patient location, and providing standardized trainee education are low-cost, generalizable strategies that other hospitals can adopt to improve timely rehabilitation.

**Funding for the study:**

N/A.

